# Circulating microRNA and automated motion analysis as novel methods of assessing chemotherapy-induced peripheral neuropathy in mice

**DOI:** 10.1371/journal.pone.0210995

**Published:** 2019-01-24

**Authors:** Qinghai Peng, Jordan Mechanic, Ahmed Shoieb, Ingrid D. Pardo, Laura Schaevitz, Judith Fenyk-Melody, Allison Vitsky, Magalie Boucher, Chris Somps, Jon C. Cook, Chang-Ning Liu

**Affiliations:** 1 Drug Safety Research & Development, Pfizer Worldwide Research & Development, La Jolla, California, United States of America; 2 Vium Inc., Milpitas, California, United States of America; 3 Drug Safety Research & Development, Pfizer Worldwide Research & Development, Groton, Connecticut, United States of America; 4 Comparative Medicine, Pfizer Worldwide Research & Development, Cambridge, Massachusetts, United States of America; 5 Drug Safety Research & Development, Pfizer Worldwide Research & Development, Cambridge, Massachusetts, United States of America; University of Florida, UNITED STATES

## Abstract

Chemotherapy-induced peripheral neuropathy (CiPN) is a serious adverse effect in the clinic, but nonclinical assessment methods in animal studies are limited to labor intensive behavioral tests or semi-quantitative microscopic evaluation. Hence, microRNA (miRNA) biomarkers and automated in-life behavioral tracking were assessed for their utility as non-invasive methods. To address the lack of diagnostic biomarkers, we explored miR-124, miR-183 and miR-338 in a CiPN model induced by paclitaxel, a well-known neurotoxic agent. In addition, conventional and Vium’s innovative Digital Vivarium technology-based in-life behavioral tests and postmortem microscopic examination of the dorsal root ganglion (DRG) and the sciatic nerve were performed. Terminal blood was collected on days 8 or 16, after 20 mg/kg paclitaxel was administered every other day for total of 4 or 7 doses, respectively, for plasma miRNA quantification by RT-qPCR. DRG and sciatic nerve samples were collected from mice sacrificed on day 16 for miRNA quantification. Among the three miRNAs analyzed, only miR-124 was statistically significantly increased (5 fold and 10 fold on day 8 and day 16, respectively). The increase in circulating miR-124 correlated with cold allodynia and axonal degeneration in both DRG and sciatic nerve. Automated home cage motion analysis revealed for the first time that nighttime motion was significantly decreased (P < 0.05) in paclitaxel-dosed animals. Although both increase in circulating miR-124 and decrease in nighttime motion are compelling, our results provide positive evidence warranting further testing using additional peripheral nerve toxicants and diverse experimental CiPN models.

## Introduction

Chemotherapy-induced peripheral neuropathy (CiPN) is a serious and commonly seen adverse effect in patients treated with chemotherapeutic agents, including platinum-based agents, taxanes, vinca alkaloids, thalidomide, bortezomib and ixabepilone. For paclitaxel, cisplatin and oxaliplatin, estimates for the occurrence of CiPN are as high as 70–90% [1–5]. The cost of CiPN on health systems is significant [6]. CiPN patients report a reduced quality of life [7], [8] and disruption of physical abilities [7]. CiPN can also lead to dose reduction of chemotherapeutic drugs or the possible cessation of treatment [9, 10]. Since the exact pathophysiology of CiPN has not been elucidated [11], treatment of this condition remains a challenge [12, 13].

In drug discovery and development, evaluation of the peripheral nervous system (PNS) in nonclinical toxicity studies is currently mandatory for predicting clinical neurotoxicity. Histopathology is currently the most commonly used method in assessing for the presence of axonal degeneration or demyelination of the peripheral nerves and, sometimes, neuronal degeneration in the dorsal root ganglia (DRG) in prospective neurotoxicity studies [14]. Although common, this terminal endpoint is labor intensive, semi-quantitative, time-consuming and insensitive. Clinically popular electrophysiological measurements of nerve conduction velocity are also used in certain circumstances, but they have methodological limitations in nonclinical toxicity studies [15, 16]. Mechanical or thermal allodynia is a typical symptom of peripheral neuropathy in both humans and animals and is commonly assessed using von Frey filaments [17]. However, for correct implementation, careful animal handling, application of a series of filaments and sophisticated measurement skills are needed [18]. Therefore, there is a need for more consistent, automated, and clinically relevant methods, such as translatable circulating biomarkers, to generate comparable and reproducible data for assessing for the presence of CiPN in nonclinical animal models.

MicroRNAs (miRNAs) appear in extracellular fluid once cellular membrane integrity is compromised. Global changes in miRNA expression in DRG have been reported in a variety of peripheral nerve injury models, including nerve transection [19–23] and spinal nerve ligation [24–26]. Nerve injury has also been shown to cause changes in miRNA expression in the sciatic nerves, a phenomenon that is postulated to reflect miRNA expression in the axons of DRG neurons and/or Schwann cells [20, 27]. miRNA expression levels in plasma were also reported to be altered in several nerve injury animal models [28]. However, the miRNA changes in the circulating blood or DRG in CiPN animal models remain unstudied.

MicroRNA-124 (miR-124) is highly expressed in brain, at levels higher than other tissues [29, 30]. Mature miR-124 is wholly homologous in mice, rats, and human, and has been reported to participate in neurodegeneration, alcohol/cocaine neuroadaptation, synapse morphology, neurotransmission, long-term potentiation, and neurodevelopment, as well as myeloid cell function, hematopoiesis and chronic stress [31]. When miR-124 is aberrantly expressed, it contributes to pathological conditions involving the central nervous system (CNS). It has also been shown to be promising as a diagnostic and prognostic indicator of CNS disorders, such as stroke [32]. But in regard to the PNS, particularly with respect to CiPN, research in the role and expression of the miRNAs is relatively scarce. In an *in vitro* study, microRNA-338 was shown to enhance axonal regeneration and myelination in neurons [33].

The behavioral changes in CiPN animals have often been observed and recorded during daytime [34–41], but nighttime behavior, particularly motility, has not been intensively evaluated due to technical challenges. In recent years, gait analysis has been used in evaluating the behavioral changes in the animal model of CiPN. However, gait data are typically acquired during the day and the technologies acquire just seconds to minutes of time, which limits the sensitivity for detecting CiPN. Here we used a new technology that enables continuous acquisition and streaming of activity and motion data during the nighttime, when rodents are naturally more active and detection of changes could be more sensitive.

In the work reported here, we explored miRNA biomarkers and automated home cage motion tracking in mice treated with paclitaxel, a severe neurotoxic agent [42], and assessed their potentials as early CiPN biomarkers. Other chemotherapy agents, such as cisplatin and oxaliplatin, were not tested in this study.

## Materials and methods

### Experimental design

Two experiments were conducted independently; one within Pfizer and the other within Vium animal facilities. In both experiments, CiPN was induced in mice by intraperitoneal (i.p.) administration of paclitaxel (Sigma-Aldrich, St Louis, MO) at 20 mg/kg, in a vehicle of 0.1 mg of cremophor EL:ethanol (1:1) in 100 ml sterile saline, once every other day for 4 or 7 doses. Paclitaxel solutions were diluted just prior to dosing and injected at a volume of 10 ml/kg [43].

The first experiment was conducted at Pfizer. Adult male C57BL/6 mice (Jackson Labs [JAX], Bar Harbor, ME) weighing 24–29 g at the start of the study, were individually housed in plastic cages on paper bedding, in animal rooms with controlled temperature (20–26°C) and humidity (30–70%), and a 12/12 h light/dark cycle. Animals were administered vehicle (n = 8) or paclitaxel (n = 8) every other day for a total of 7 doses. In order to assess general toxicity and potential mortality, animals in both groups were checked daily, while body weight and food consumption were measured every other day. On day 16 the animals were euthanized via an overdose of isoflurane and tissues were terminally collected. Two additional groups, each with 5 mice, were treated every other day with vehicle or paclitaxel for 4 doses and plasma samples were obtained on day 8 for miRNA biomarker measurements.

In the second experiment, conducted at Vium, two groups of 8 seven-week old male C57BL/6 mice (Charles River Laboratory[CRL], Hollister, CA) were tested. Mice were housed individually in Smart Cages on corn cob bedding (Anderson’s 1/8 inch cob; Maumee, OH) in animal rooms with controlled temperature (20–26°C) and humidity (30–70%), and a 12/12 h light/dark cycle. Mice were administered injections of either vehicle or paclitaxel every other day for a total of 7 doses. Mice were monitored 24 hours per day using automated motion, breathing rate, and running wheel metrics generated by the Vium Digital Platform [44]. On day 16, mice were euthanized with an overdose of isoflurane. Terminal blood and DRGs from L3/L4 and sciatic nerves were harvested for biomarker and histologic evaluation at Pfizer.

All activities involving mice were carried out in AAALAC accredited facilities and in accordance with federal, state, local and institutional guidelines governing the use of laboratory animals in research. Study protocols were reviewed and approved by Pfizer and Vium Institutional Animal Care and Use Committees (IACUCs) in compliance with the Guide for the Care and Use of Laboratory Animals, 8th Edition (National Research Council, 2011).

### Vium digital vivarium technology

Vium Digital Smart Cages were outfitted with sensors that stream animal data and environmental conditions 24 hours a day, 7 days a week to a secure cloud-based data infrastructure [44]. The Vium Digital Platform obtained and displayed the following information continually in near real-time: (1) conducted procedures with corresponding times; (2) data analytics on motion, breathing rate, and running wheel activity; and (3) verification of illumination. This study used the validated Vium Motion metric, Vium Breathing Rate metric, and Vium Running Wheel metric.

### Conventional behavioral assessments

Cold allodynia was assessed on days -1, 5, 12, and 15 in both JAX and CRL mice. Cold allodynia of the tail was assessed in mice using the method described by Authier and colleagues [35]. Briefly, the tail of the mouse was immersed approximately 1 cm below the surface of 1000 ml of ice-cold water (~80% ice and ~20% water at ~0° C). The latency to the first flick response was recorded with a cutoff time of 15 sec [45]. The trial was repeated 3 times with an interval of greater than 5 minutes between each cold stimulus. Average values of the three trials were used for analysis.

### Plasma and tissue RNA extraction and miRNA measurement

On both JAX and CRL mice, plasma RNA was extracted as previously described (37) and tissue RNA extraction was conducted on segments of the sciatic nerve (0.3–0.5 cm length) and the DRGs. Tissues were dissected under a dissection microscope, placed in Ambion RNAlater solution (Cat #: AM70201, Thermo Fisher Scientific, Waltham, MA) at 4°C overnight, and then transferred to -20°C freezer until further tissue processing. Tissues were transferred into 2 ml tubes containing 1 ml of Qiazol and one stainless steel bead (5 mm, cat #: 69984, Qiagen, Venlo, Netherlands) and exposed to frequency of 25 Hz Qiagen TissueLyser II for 10 minutes until a homogenous lysate was formed without visible particles. The total RNA was extracted from the tissue lysate or 100 ul of plasma using Qiagen’s miRNeasy kit according to the manufacturer’s protocol. One hundred ng of the purified total RNA from the tissues or 5 ul of total RNA purified from plasma was then subjected to quantitative real time PCR (RT-qPCR). Quantification of miRNAs was performed in duplicate in the final PCR reactions. All reactions were run on a ViiA 7 Real Time PCR System (Thermo Fisher Scientific) using the following conditions: 95°C for 10 min, followed by 40 cycles at 95°C for 5 seconds, and then 60°C for 30 seconds. Data is presented as relative copy number ± standard error of the mean (SEM). Standard curves were generated using synthetic RNA oligos. Reference RNAs such as endogenous miRNAs of miR-92, miR-181 and miR-192, and exogenous spiked-in Cel-miR-39 were detected in plasma samples while endogenous U6 snRNA was quantified with tissue samples for normalization purposes [46]. [46]. In a pilot rat study, miRNAs from various nervous system and non-nervous system tissues were extracted, purified and subjected to RT-qPCR. The copy numbers in tissue expression were compared. Three peripheral nerve-enriched microRNAs (miR-124, miR-183 and miR-338) were identified in this global profiling experiment.

#### Histological evaluation of DRG and peripheral nerve

On both JAX and CRL mice, DRGs (L3 –L5), sciatic nerve, tibialis anterior muscle, and gastrocnemius muscle samples were collected and fixed in 4% methanol-free formaldehyde. Samples of sciatic nerve were carefully dissected from the proximal aspects of the thigh to the knee joint just proximal to the point of division into the common peroneal, tibial, and sural nerves; the middle segment of this sample was collected. Tissues were processed, embedded in paraffin, sectioned longitudinally at 5 μm thickness and stained with haemotoxylin and eosin (H&E) as described by other investigators [47] for microscopic examination. The nerve and DRG sections were evaluated under light microscopy and images were scanned using Leica Aperio AT2 whole slide digital scanner and acquired using ImageScope viewing software (Leica Biosystems, Vista, CA). Microscopic lesions were graded on a scale of 1 to 5 as minimal, mild, moderate, marked, or severe.

### Statistical analysis

All the results were reported as mean ± SEM. The data were statistically analyzed by Student’s *t*-test or two-way analysis of variance (ANOVA) followed by Dunnett’s or Bonferroni’s post hoc test using Graphpad Prism 6 software (La Jolla, CA). P values of less than 0.05 (*) were considered statistically significant.

## Results and discussion

### General toxicity of paclitaxel

**Fig 1:**
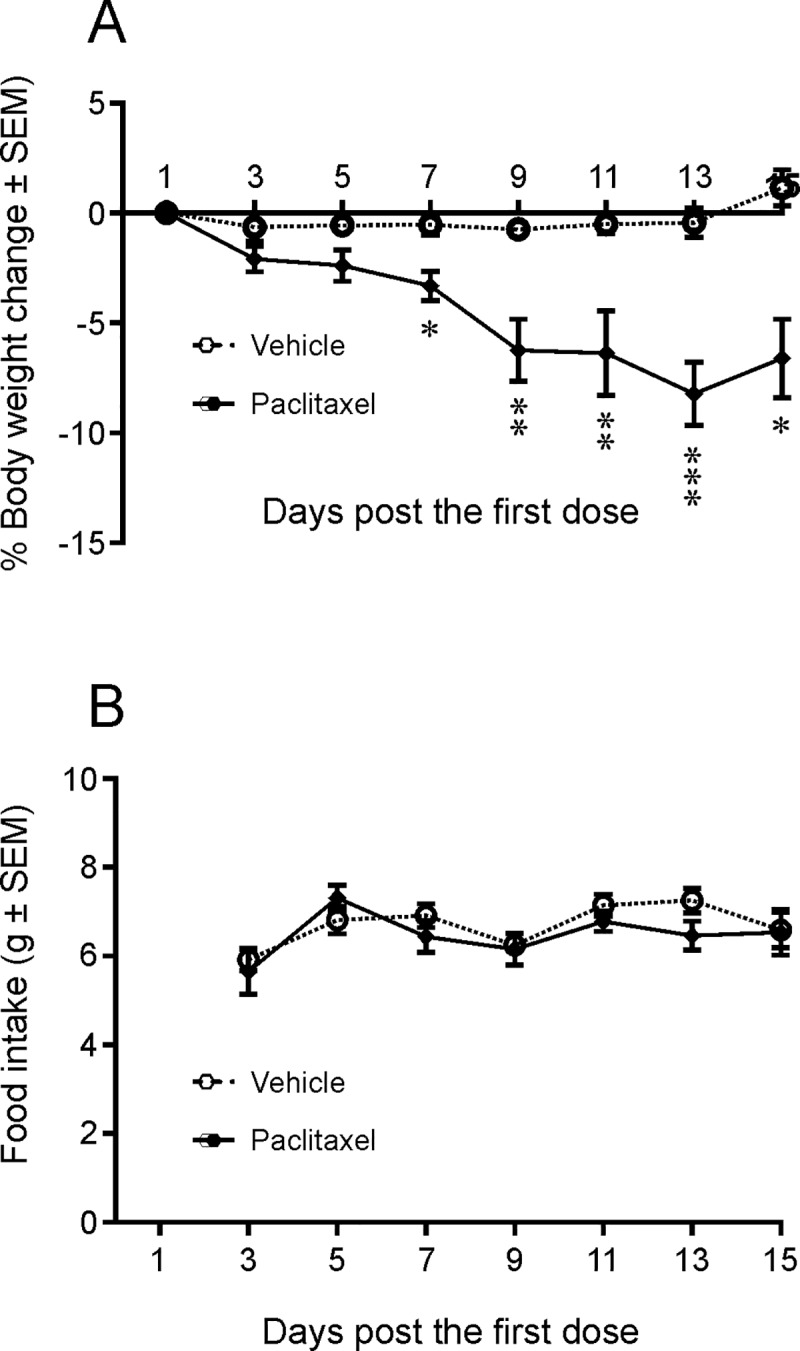
Body weight and food consumption changes following paclitaxel administration in JAX mice. Body weights were measured every other day from the first dosing day and food intakes were measured every other day from day 3 following the first dose. Open symbols and dashed lines represent the values from vehicle control animals and filled symbols and solid lines represent paclitaxel-dosed animals. The group average body weights (A) was significantly (*P < 0.05; **P < 0.01; ***P < 0.001, two-way ANOVA) decreased in the paclitaxel treated mice, as compared to the vehicle treated group [n = 8]). There were no changes in food consumption (B) between the mice dosed with paclitaxel and those dosed with vehicle (P > 0.05).

Paclitaxel-dosed JAX mice showed significant weight loss starting from day 7 (-3.3% vs. -0.5% in vehicle-dosed animals, P < 0.05, two-way ANOVA), peaking on day 13 (-8.2% vs. -0.4% in vehicle-dosed animals, P < 0.001). The mice in the vehicle-treated group showed stable body weight (Fig 1A, S5 File). Food intake was not altered in either group (Fig 1B). None of the mice treated with paclitaxel manifested obvious clinical signs of morbidity during the in-life phase. In CRL mice, body weight failed to show a statistically significant change in paclitaxel treated mice (P > 0.05, S3 and S4 Files). All control mice (8/8) survived until study end (day 16) while only 7/8 (87.5%) of paclitaxel mice survived until study end. A single paclitaxel treated mouse did not reach study endpoint and was found dead on day 12. Gross necropsy during blood and tissue collection revealed the presence of fluid in the abdominal cavity and duodenum, possibly due to the effect of paclitaxel on intestinal mucosa [48].

### Cold allodynia

**Fig 2:**
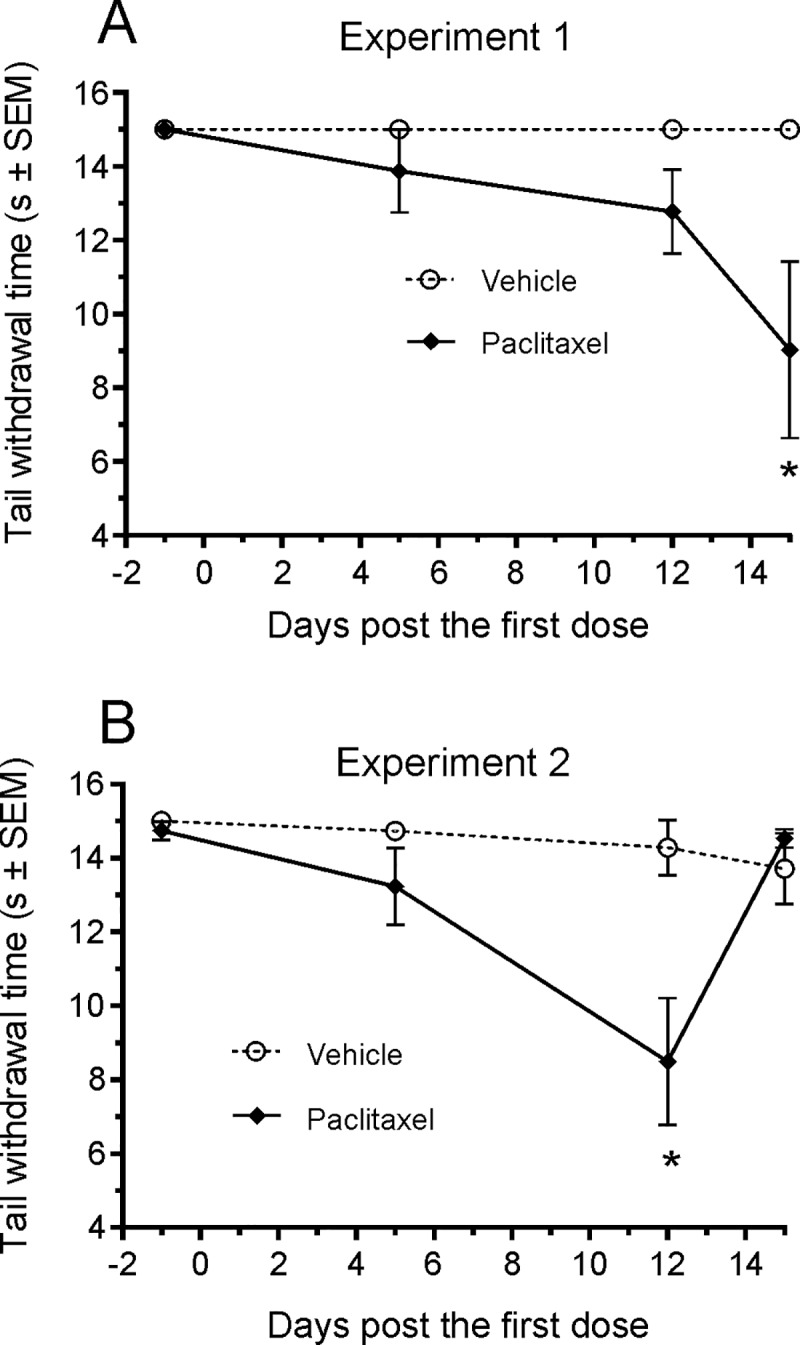
Cold allodynia. (A) The latencies of tail flick (time from cold water (~0°C) stimuli applied to the tip of tail and a flick response) are shown as a function of time following the first dose of paclitaxel or vehicle. All the vehicle control JAX mice had latencies greater than the 15 sec monitoring cut-off time. Five of 8 JAX mice dosed with paclitaxel at 20 mg/kg had reduced latencies on days 12 and 15 and the reduced grouped latency was statistically significant on day 15 (*P < 0.05, Two-way ANOVA). (B). The difference in the latencies between paclitaxel treated (n = 8) vs vehicle treated CRL mice (n = 8) was statistically significant by day 12.

None of the vehicle-treated JAX mice responded to the cold stimuli within the 15 sec cut-off time when tested predose (day-1) and on days 5, 12, and 15. On day 5 after paclitaxel dosing, one paclitaxel-treated animal responded with a tail flick latency of less than 15 sec. On days 12 and 15 responders to cold stimuli increased to 63% (5 out of 8 mice). On day 15, the average latency of cold-induced tail flick of the paclitaxel-dosed mice was significantly shorter compared to the vehicle treated mice (P < 0.05, Fig 2A). Likewise, in CRL mice, there was a statistically significant reduction in the cold-allodynia latency in the paclitaxel-treated animals on day 12 (P < 0.05, Fig 2B).

### Microscopic evaluation of DRG and sciatic nerve

**Fig 3:**
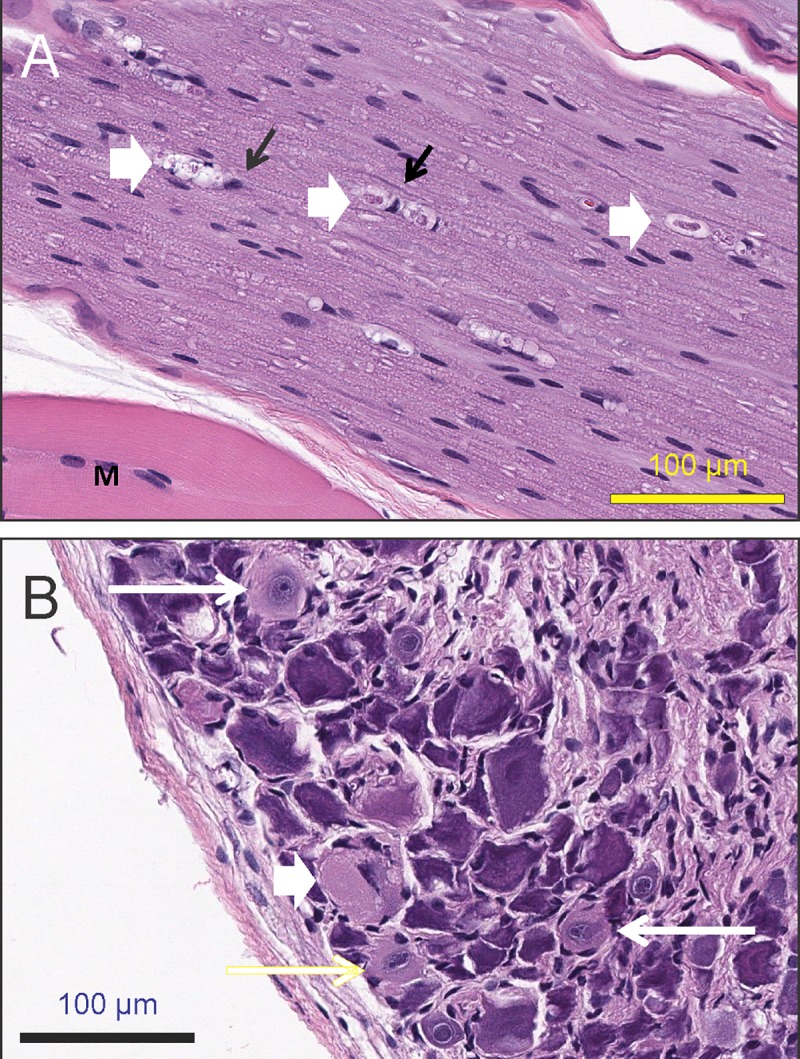
Microscopic findings of the nerve and DRG in JAX mice. (A) Nerve fiber degeneration and dilation of myelin sheaths containing eosinophilic granular material (digestion chambers, short white arrows) which are surrounded by enlarged Schwann cells (long black arrows). M: Skeletal muscle. (B) Neuronal degeneration and chromatolytic neurons. Note the dissolution of Nissl substance and displacement of the nucleus to the periphery of the cell (Short white arrow), and neurons undergoing early chromatolytic change. Note the eosinophilic granular appearance of the cytoplasm and accumulation of the Nissl substance near the enlarged nuclei (long white and yellow arrows).

In JAX mice, minimal-to-mild axonal degeneration was present in the sciatic nerve, spinal nerve roots of the right L4 and L5 DRGs, and in nerves innervating the left gastrocnemius and/or tibialis anterior muscles (Fig 3A) of the animals dosed with paclitaxel but was not observed in the vehicle-dosed animals. Minimal chromatolysis, characterized by the dissolution of the Nissl bodies (rough endoplasmic reticulum, RER) in the soma of neuron, was present in some neurons of the right L4 DRGs (Fig 3B). In CRL mice, minimal and mild axonal degeneration was present symmetrically in the right and left sciatic nerve samples of 2 (of 8) animals dosed with paclitaxel. In the samples from the same 2 animals, minimal chromatolysis in DRGs was observed symmetrically. Group incidences of the microscopic findings are presented in Tables 1 and 2.

**Table 1:** Group incidences (with severities) of microscopic findings in JAX mice.

Tissue	Finding		Group	
			Vehicle	Paclitaxel
**Sciatic nerve**	Axonal degeneration	Examined total	8	8
		Minimal (Grade 1)	-	4
		Mild (Grade 2)	-	3
**L4 DRG (right**)	Chromatolysis	Examined total	7*	8
		Minimal (Grade 1)	-	4
**Spinal nerve root**	Axonal degeneration	Examined total	7	8
		Minimal (Grade 1)	-	5
**L5 Dorsal root**	Degeneration	Examined total	7	7
		Minimal (Grade 1)	-	3
**Gastrocnemius muscle**	Axonal degeneration	Examined total	8	8
		Minimal (Grade 1)	-	3
**Tibialis muscle**	Axonal degeneration	Examined total	8	8
		Minimal (Grade 1)	-	1

Grade 1 = minimal. Grade 2 = mild.

- = No finding present.

* = One ganglion was damaged during tissue preparation.

**Table 2:** Group incidences (with severities) of microscopic findings in CRL mice.

Tissue	Finding		Group	
			Vehicle	Paclitaxel
**Sciatic nerve (left)**	Axonal degeneration	Examined total	8	8
		Minimal (Grade 1)	-	1
		Mild (Grade 2)	-	1
**Sciatic nerve (right)**	Axonal degeneration	Examined total	8	8
		Minimal (Grade 1)	1	1
		Mild (Grade 2)		1
**L4 DRG (left)**	Chromatolysis	Examined total	8	8
		Minimal (Grade 1)	-	2
**L4 DRG (right)**	Chromatolysis	Examined total	8	8
		Minimal (Grade 1)	-	2

- = No finding present.

Grade 1 = minimal. Grade 2 = mild

### Enrichment of miRNAs in peripheral nervous tissue

**Fig 4:**
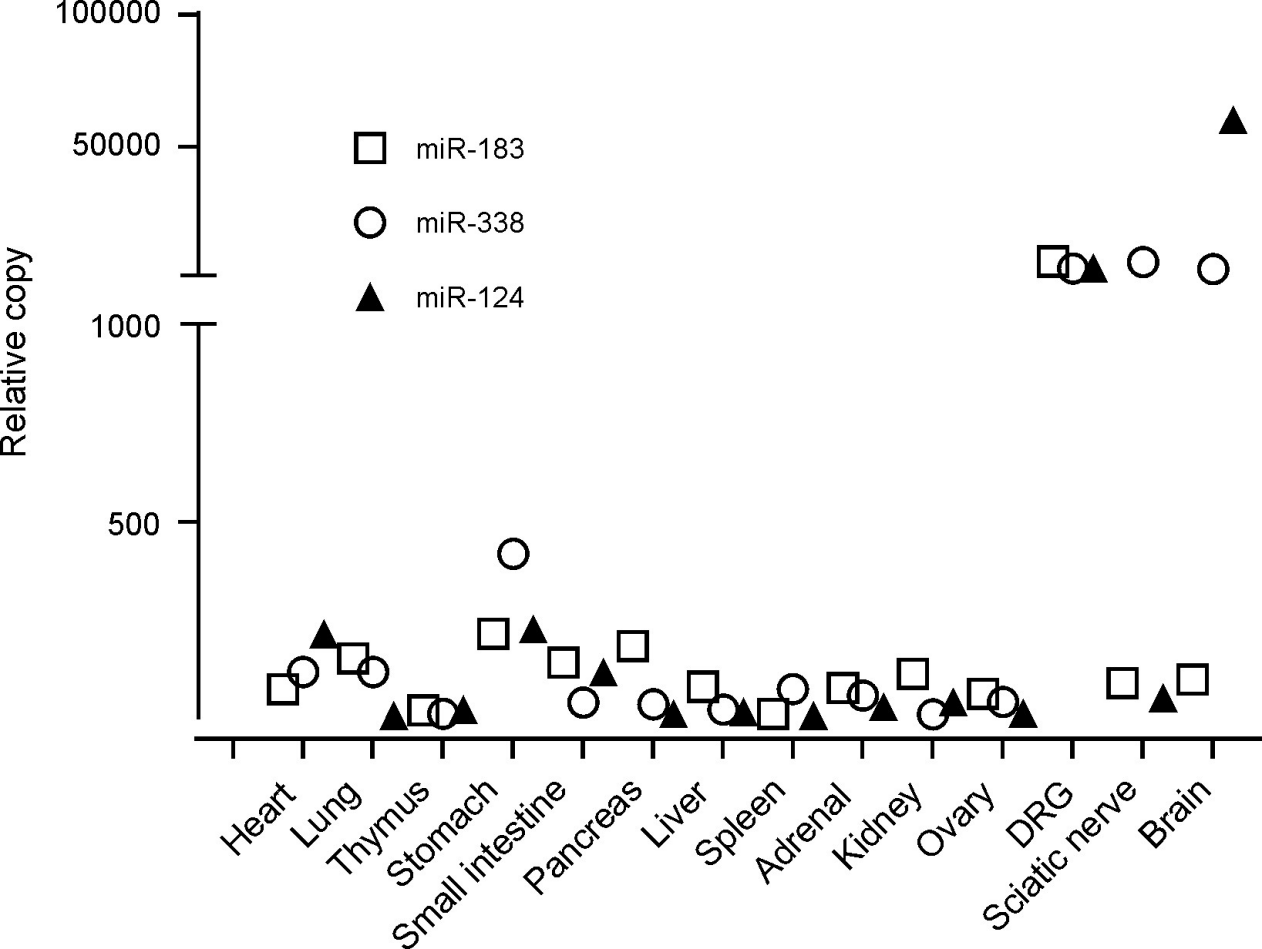
miRNAs enriched in peripheral nerve tissues. MiR-124, miR-183 and miR-338 expression levels were over 20 fold higher in DRG and sciatic nerve tissues compared to other non-nerve tissues and organs.

Total RNA of 100 ng from various tissues were extracted, purified and subjected to RT-qPCR. Copy number of each miRNA was determined as expression level and compared. miR-124, miR-183 and miR-338 were identified to be relatively enriched in peripheral nervous tissues. The differential expression of the 3 miRNAs was 20-fold higher than the mean of other non- nerve tissues and organs by global miRNA profiling in rat in RT-qPCR platform analysis (Fig 4, S1 File). However, there was not much of an expression difference between CNS and PNS for miR-338. miR-124 was more enriched in brain than PNS and miR-183 was more enriched in DRG.

### Changes in miRNA level in blood

**Fig 5:**
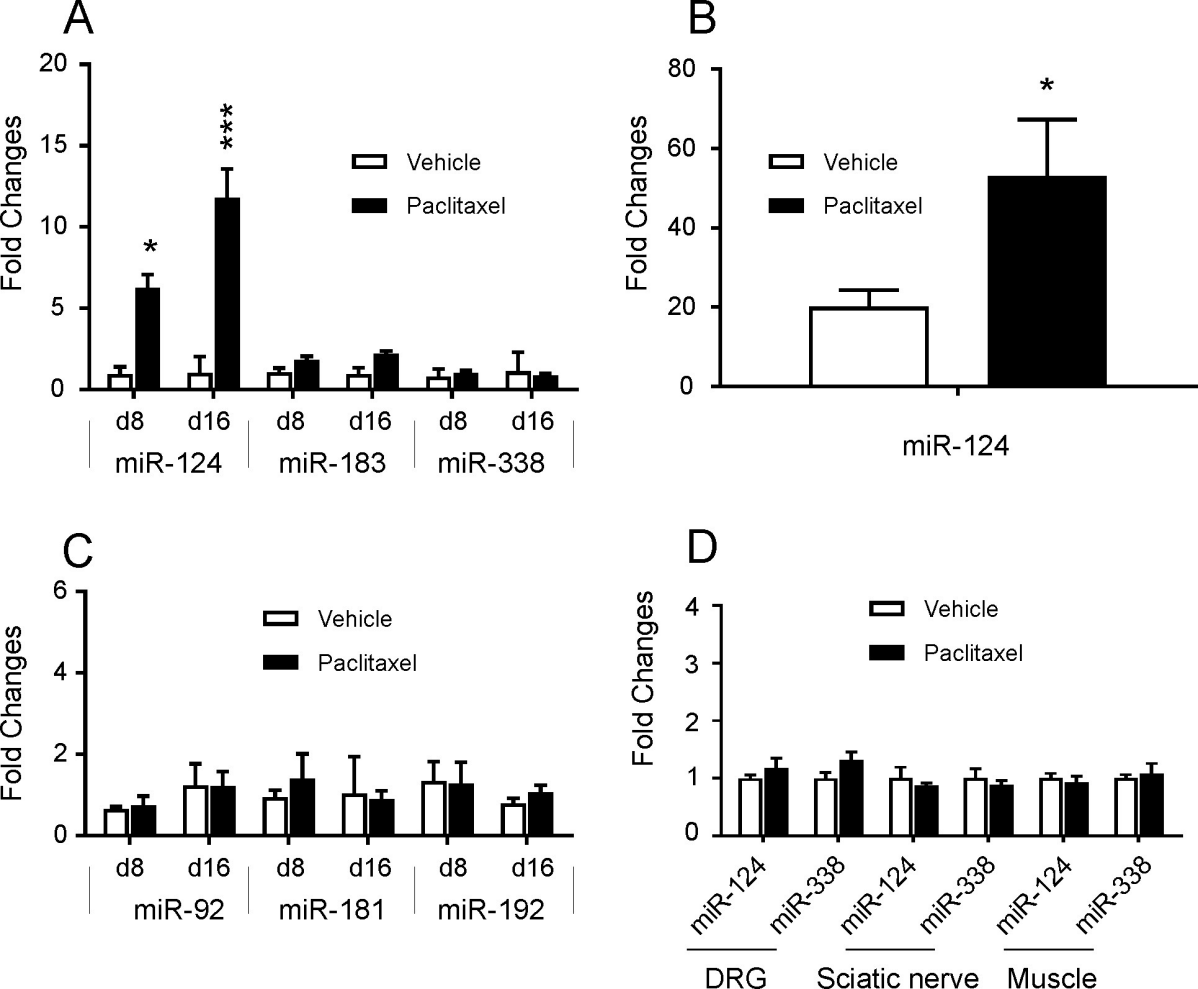
MiRNA changes in the plasma and tissues of mice. (A) In JAX mice, circulating miR-124 on day 8 (P < 0.05, n = 5) and day 16 (P < 0.001, n = 8) were increased significantly with paclitaxel treatment compared with vehicle control groups. Circulating miR-183 and miR-338 were relatively steady in the plasma of the paclitaxel-dosed animals (P > 0.05) on day 8 or day 16 compared to the vehicle controlled group. (B) In CRL mice, the average miR-124 level in the paclitaxel-treated group was significantly increased (P < 0.05) on day 16, compared to the vehicle control group. (C) Endogenous miR-92, miR-181 and miR-192 were similar between paclitaxel-dosed JAX mice and the vehicle controlled group. (D) No significant alteration in the endogenous levels of miR-124 or miR-338 was observed in the DRG, sciatic nerve or skeleton muscle between paclitaxel treated group and vehicle control JAX mice.

In JAX mice, miR-124 showed a statistically significant increase in the plasma of the mice treated with paclitaxel on day 8 (P < 0.05, n = 5) and day 16 (P < 0.001, n = 8) compared with vehicle control groups (Fig 5A, S2 File). There was no difference in plasma miR-183 or miR-338 levels of paclitaxel-treated animals (P > 0.05) on day 8 or day 16 (Fig 5A, S2 File) compared with those samples collected from the vehicle controll JAX mice. Notably, circulating miR-124 was also significantly increased in CRL mice on day 16 (P < 0.05), although the magnitude of increase was not as high as those observed in JAX mice (Fig 5B, S2 File).

### Changes in expression of miRNAs in the nervous tissue

No difference in miR-124 or miR-338 expression was observed in the DRG, sciatic nerve or skeletal muscle between tissues from paclitaxel treated JAX mice and vehicle control group animals (Fig 5D, S2 File).

### Motion changes

**Fig 6:**
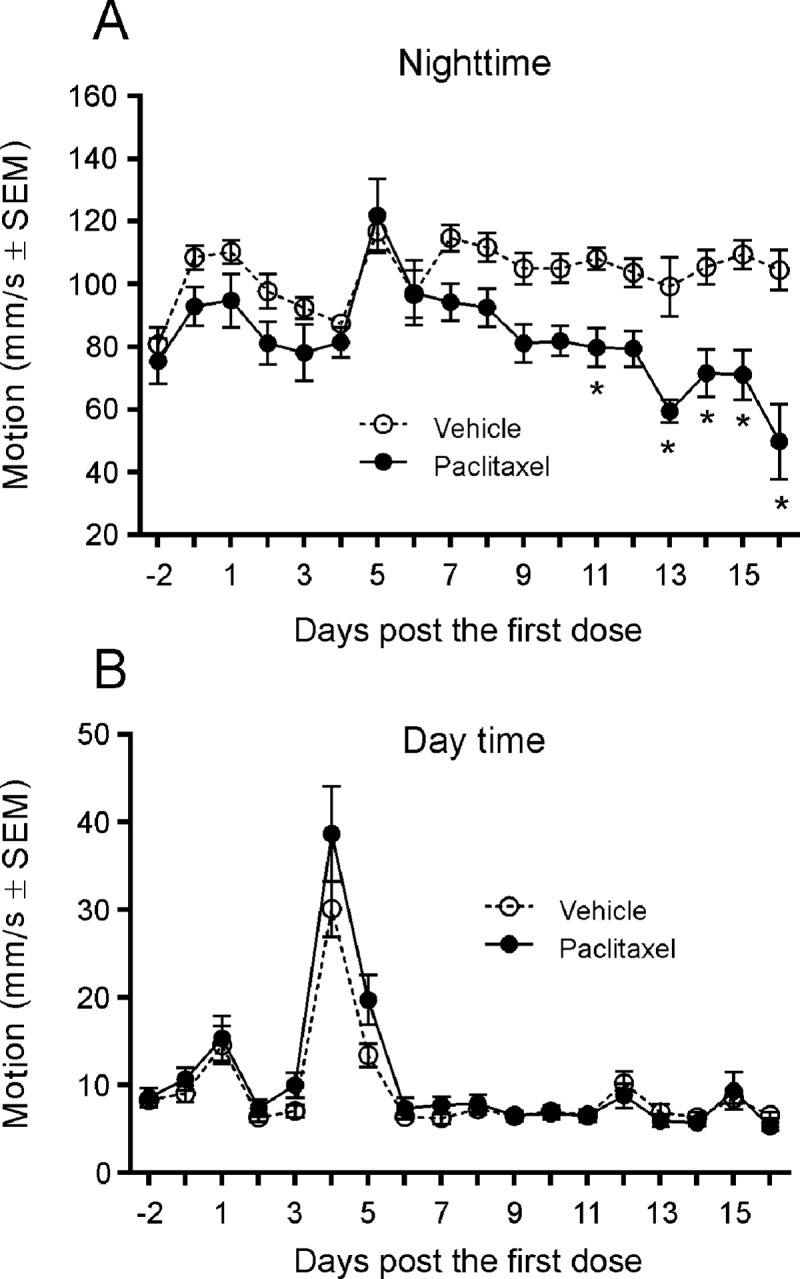
**Motion changes in night time (A) and day time (B**). Compared with the vehicle control animals, paclitaxel-dosed CRL mice showed decreases in nighttime motion on nights 11, 13–16 (A) but did not show changes in daytime motion (B). The peak in (B) indicates cage change on day 4. *, P < 0.05 vs. vehicle by Bonferonni’s test.

In CRL mice, the nighttime Motion metric demonstrated a significant interaction between treatment and study time (P ≤ 0.001) (Fig 6A). Follow-up pairwise comparisons revealed that paclitaxel-treated mice had statistically significant lower nighttime motion compared to controls on nights 11, and 13–16 (P < 0.05). For daily motion, however, there were no statistically significant main effects of paclitaxel treatment (Fig 6B).

In the present study, we demonstrated a statistically significant increase in a circulating miRNA, as well as behavioral and motion changes in two experiments of mice dosed with the commonly used chemotherapeutic agent paclitaxel (cisplatin or oxaliplatin was not tested here). For the first time, we detected increased circulating miR-124 and decreased nighttime motion in animals with CiPN, both of which can be readily incorporated into routine nonclinical rodent toxicology studies.

Paclitaxel, one of the most commonly used chemotherapeutic agents, has been reported to cause severe neurotoxicity in both the clinic as well as in animals commonly used in nonclinical investigations, such as mice [41, 49, 50]. JAX mice treated with paclitaxel at 20 mg/kg/day lost body weight immediately after dosing and continued to lose weight throughout the treatment period, particularly after day 7, the day on which the decrease in weight first became statistically significant (P < 0.05–0.001). The weight loss in these animals was unaccompanied by decrease in food intake (Fig 1B), which suggests that the weight loss in these animals was unrelated to this parameter. Paclitaxel-treated CRL mice did not demonstrate weight loss. Although this discrepancy might be related to the difference in the sources of animals (Jackson Lab vs. Charles River Laboratories) and the food provided in the two different vivaria, prior studies have also shown an absence of body weight change in C57BL/6 mice following paclitaxel administration at 4 mg/kg [41] or 60 mg/ kg [49]. Overall, this data suggests that body weight loss is not a good indicator of CiPN in mice. Despite no consistent weight loss observed in CRL mice, microscopic lesions were observed in the DRG as well as in the sciatic nerve, either close to the DRG or near the axon terminals in the skeletal muscles, in JAX and CRL mice treated with paclitaxel. In the PNS, paclitaxel has been shown to interfere with the normal breakdown of microtubules resulting in nerve fiber degeneration and/or neuronal chromatolysis [51], [52]. Our findings consisted of minimal-to-mild nerve fiber degeneration in the sciatic nerve and minimal neuronal chromatolysis in DRG neurons, which is consistent with the above cited literature. Ultrastructural observation on microtubules in DRG neurons and axons could provide more molecular links with the endpoints we measured, and would be included in future studies. In addition, cold allodynia was observed in some animals 1–2 weeks following paclitaxel administration. Taken together, these findings demonstrate the presence of peripheral neuropathy in some, if not all, animals in both experiments conducted.

Nerve tissue-enriched miR-124, miR-183 and miR-338 were evaluated as biomarker candidates in the paclitaxel-induced peripheral neuropathy model in mice. Although plasma miR-124 was shown to be significantly increased on day 8 (JAX mice) and day 16 after paclitaxel administration in JAX and CRL mice, plasma levels of miR-183 and miR-338 in the plasma remained unchanged. miR-124 is one of the multiple functional miRNAs associated with neuronal cell activity. Its increase in the plasma has been reported in CNS diseases such as stroke [32]. Previous studies have demonstrated an absence of CNS penetration of paclitaxel [53]. Therefore, it’s plausible to assume that the paclitaxel-associated increases of circulating miR-124 observed in our investigations may be connected with a peripheral effect on nerve tissue rather than an effect on the CNS. Light microscopic findings confirmed the presence of peripheral neuropathy that correlated with the increases of circulating miR-124. It was unclear why only circulating miR-124, but not miR-183 and miR-338, was increased following paclitaxel treatment in our study despite miR-338 being quite enriched in DRG and more enriched than miR-124 in sciatic nerve. This may be due to some unknown signaling pathways underlying paclitaxel toxicity. For instance, Lin and co-workers [54] observed that mechanical nerve injury-induced mechanical allodynia is significantly correlated with the decreased expression of miR-183 in DRG neurons. miR-338 has been found as an important regulator of oligodendrocyte differentiation [55] and at this time it is not clear whether miR-338 plays a similar role in peripheral Schwann cells. Alternately, there may be active secretion of miR-124 but not the other two miRNAs evaluated following paclitaxel administration. It would be interesting to test miR-124 changes following chemotherapy in cancer patients who may have genomic alteration compared to normal controls. Automated activity metrics, including spontaneous motion and running wheel activity, detected behavioral activity differences between paclitaxel treatment and vehicle control groups. Compared with controls, paclitaxel-treated CRL mice showed lower motion levels and spent a smaller percentage of time on the running wheel during the dark cycle when rodents are normally active. These changes were observed as early as night 8 and further progressed until study end. Motion-based deficits in peripheral neuropathy mouse models have been described in the literature, including gait abnormalities during daytime, which may contribute towards reductions in both spontaneous and running wheel activity [38, 56]. Sensory polyneuropathy, characterized by increases in mechanical or thermal pain sensitivity, may also affect overall activity. Regardless of treatment group, increases in daytime activity were most apparent on days associated with coincidental stimulation following cage changing (day 4) or dosing and behavioral testing (days 1, 5, 12, and 15) (similar to an unpublished observation). In the Vium digital vivarium system, once the study design is set up prior to the study start, charts and graphs will be automatically updated as new data is collected by computers. In the current study, this process greatly reduced the potential bias introduced by humans, which is usually seen in the conventional behavioral testing.

## Conclusions

CiPN is a serious and common adverse effect in patients treated with chemotherapeutic agents for which there is a need for more consistent, automated, and clinically relevant methods to routinely assess toxicity. Plasma levels of miR-124 may be a good translatable blood biomarker for CiPN. Furthermore, automated assessment of nighttime motion levels might be good to incorporate into routine mouse toxicity studies to predict CiPN risk in a high throughput mode.

## Supporting information

S1 File PCR data for Fig 4. RT-qPCR results of miR-124, -183 and -338 in tissues.
(XLSX)

S2 File PCR data for Fig 5. RT-qPCR results of miRNAs in plasma and tissue of JAX and CRL mice dosed with paclitaxel or vehicle.
(XLSX)

S3 File Body weight comparison of CRL mice. Body weight data in percentage and graph of body weight changes in CRL mice administered paclitaxel or vehicle.
(PZFX)

S4 File Body weight data of CRL mice. Body weight raw data of CRL mice administered paclitaxel or vehicle.
(CSV)

S5 File Body weight data of JAX mice. Body weight raw data and graph of JAX mice administered paclitaxel or vehicle.
(PZFX)
